# Supplementary material for: Asymmetric coding of reward prediction errors in human insula and dorsomedial prefrontal cortex
Source: Nat Commun. 2023 Dec 21;14:8520. doi: 10.1038/s41467-023-44248-1 (PMC10739882; doi:10.1038/s41467-023-44248-1)
Supplement: Supplementary file 1 — Supplementary Information [file 41467_2023_44248_MOESM1_ESM.pdf]

Asymmetric coding of reward prediction errors in human insula and dorsomedial prefrontal cortex

*Colin W. Hoy, *David R. Quiroga-Martinez, Eduardo Sandoval, David King-Stephens, Kenneth D. Laxer, Peter Weber, Jack J. Lin, Robert T. Knight

SUPPLEMENTARY INFORMATION:

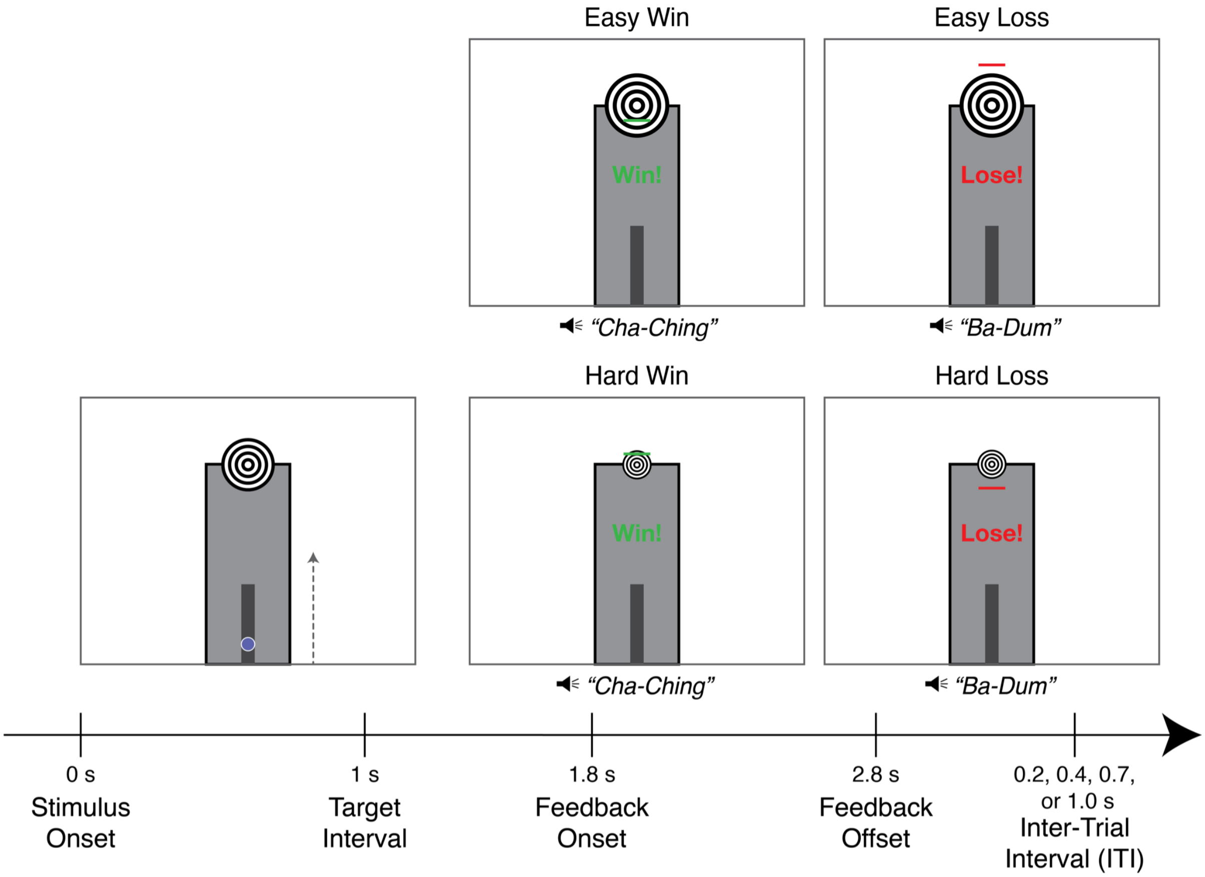

Supplementary Figure 1. ***Task version No 1.*** *Participants pressed a button to estimate the time when a ball traveling upwards hit the center of the bullseye target. The size of the bullseye cue displayed error tolerance around the 1 s target interval. Audiovisual feedback is indicated by the text "Win!" in green or "Lose!" in red, along with a tick mark of the same color marking the RT.*

Supplementary Table 1. ***Coefficients of the winning asymmetric RPE model for behavioral data analyses of the iEEG experiment.***

Significance (uncorrected p-values) was obtained using one-sided conditional Wald tests with the Kenward-Rogers approximation. 95% confidence intervals (CI) and degrees of freedom (df) are also reported. Significant p-values are highlighted in bold. Source data are provided as a Source Data file.

Coefficient	Estimate	CI (95%)	*p*	df

Intercept	0.03	[0, 0.06]	**0.029**	14.59
Previous RT	-0.75	[-0.85, -0.65]	**< 0.001**	63.31
nRPE	0.01	[-0.01, 0.02]	0.218	3884.67
pRPE	0.02	[0,01, 0.04]	**< 0.001**	3885.87
Outcome (Neutral)	-0.01	[-0.03, 0.01]	0.375	3877.43
Outcome (Loss)	0	[-0.02, 0.02]	0.856	3888.16
Previous RT * Outcome (Neutral)	0.01	[-0.14, 0.16]	0.868	3631.20
Previous RT * Outcome (Loss)	-0.27	[-0.36, -0.18]	**< 0.001**	3886.87

Supplementary Table 2. ***Coefficients of the winning asymmetric RPE model for behavioral data analyses of a previous EEG experiment.***
Significance (uncorrected p-values) was obtained using one-sided conditional Wald tests with the Satterthwaite approximation. Confidence intervals (CI) and degrees of freedom (df) are also reported. Significant p-values are highlighted in bold. Source data are provided as a Source Data file.

Coefficient	Estimate	CI (95%)	*p*	df
Intercept	0.01	[0, 0.02]	**0.002**	17240
Previous RT	-0.90	[-0.95, -0.85]	**< 0.001**	17240
nRPE	0	[-0.01, 0]	0.218	17240
pRPE	0.02	[0,02, 0.03]	**< 0.001**	17240
Outcome (Neutral)	-0.01	[-0.02, -0.01]	**< 0.001**	17240
Outcome (Loss)	0	[-0.01, 0]	0.193	17240
Previous RT * Outcome (Neutral)	0.16	[0.10, 0.21]	**< 0.001**	17240

Previous RT * Outcome (Loss)	-0.10	[-0.14, -0.06]	**< 0.001**	17240

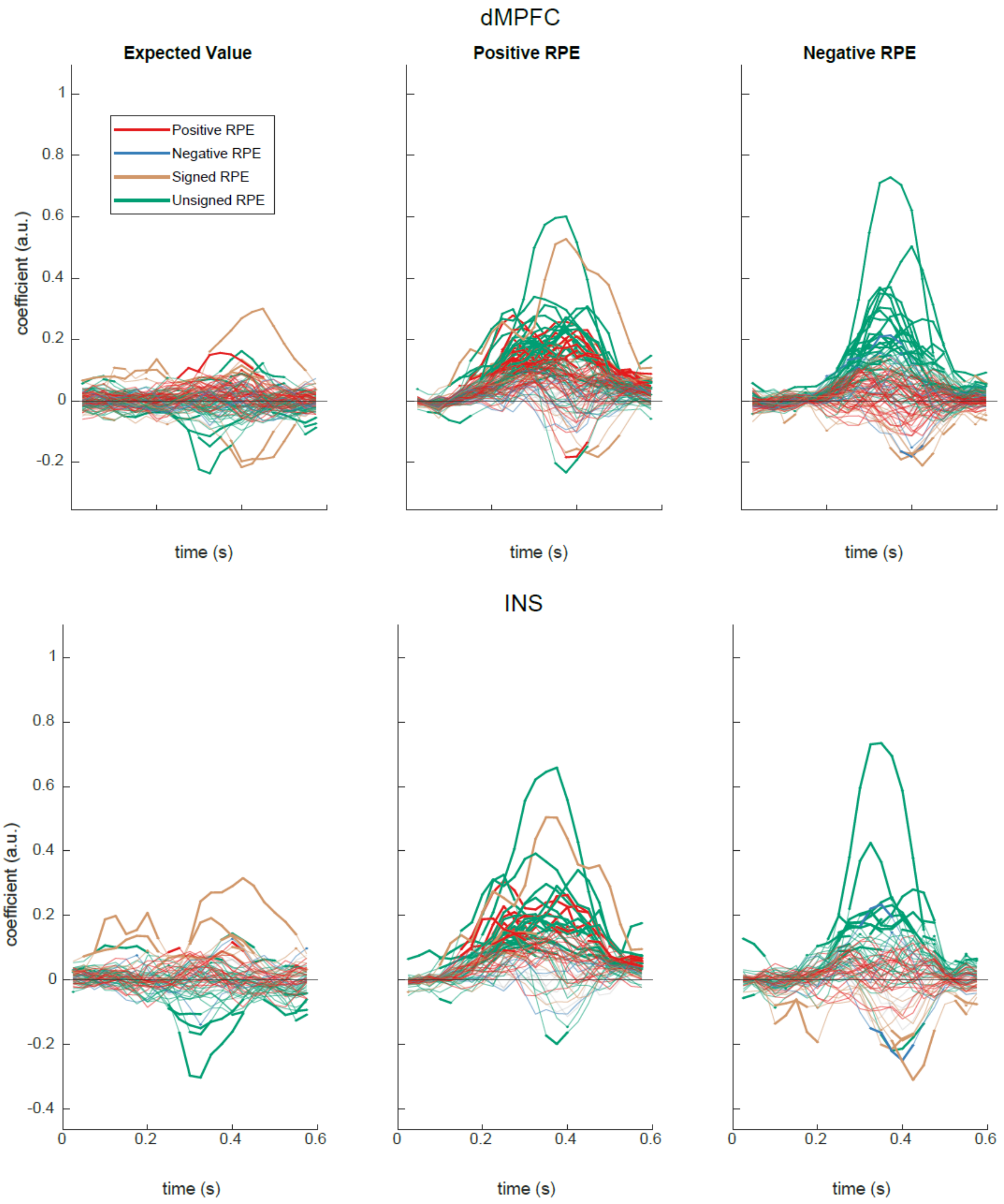

Supplementary Figure 2. ***Time course of individual channel random-effects coefficients for each of the three regressors in the asymmetric RPE model of HFA.***
Colors indicate channel category. Significant timepoints are highlighted in bold (uncorrected p<0.05 for two-sided t-tests). Source data are provided as a Source Data file.

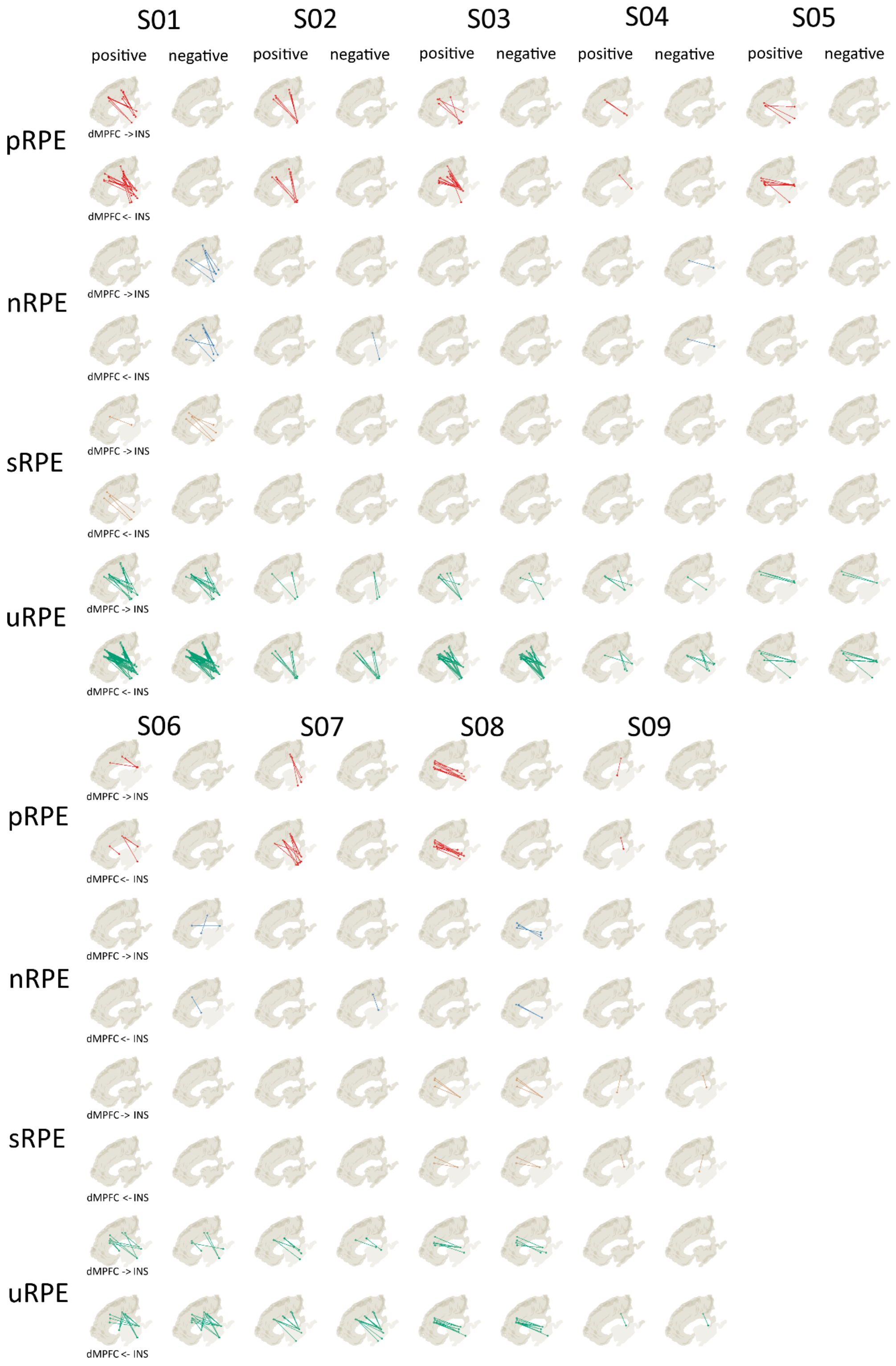

Supplementary Figure 3. ***Patient specific connectivity for different regressors (positive/negative RPE), directions of communication (INS > dmPFC, dmPFC>INS) and channel categories (pRPE, nRPE, sRPE, uRPE).***
Statistics as described in Fig. 4c. Source data are provided as a Source Data file.

Supplementary Note 1: Analysis of differences in RPE categories between anterior and posterior dMPFC for connectivity estimates

The dMPFC ROI used in the main analyses includes multiple subregions. As an initial assessment of differences across subregions, we tested whether patterns of RPE categories were different between anterior and posterior divisions of dMPFC. Using linear mixed-effects models to predict peak lags and comparing them through likelihood ratio tests, we confirm no significant differences between anterior (y MNI coordinate < 12) and posterior (y MNI coordinate ≥ 12) dMPFC connections for either negative RPE coefficients ($\chi^2(6) = 3.13$, $p = 0.79$) or positive RPE coefficients ($\chi^2(6) = 2.92$, $p = 0.81$). Similarly, there are no significant interactions between subregion and channel pair category (positive RPE coefficients: $\chi^2(24) = 10.74$, $p = 0.99$; negative RPE coefficients: $\chi^2(24) = 9.35$, $p = 0.99$).

Supplementary Note 2: Sensitivity Analyses

As shown in Supplementary Figure 4, HFA residuals were skewed towards the positive end. We therefore did a sensitivity analysis transforming HFA values into rankit as follows:

$$rankit(HFA) = \sigma(HFA)\ F^{-1}(rank(HFA) - .5) + \mu(HFA)$$

This transformation ranks the data points from low to high, centers them between integers by subtracting 0.5, then projects the ranks onto a gaussian distribution by taking the inverse of the normal cumulative distribution function (F^{-1}), and finally scales and centers the data to the standard deviation (σ) and mean (μ) of the original HFA values. As shown in Supplementary Figure 4, this transformation leads to gaussian residuals. Note that while the transformation preserves the ordering of original data points, it modifies their value. This is valid for statistical testing but makes the interpretability of coefficients difficult. Supplementary Figure 5 shows the convergence of results from original and sensitivity analyses. A similar sensitivity analysis was done for connectivity data, which exhibited heavy-tailed residuals (Supplementary Figure 6).

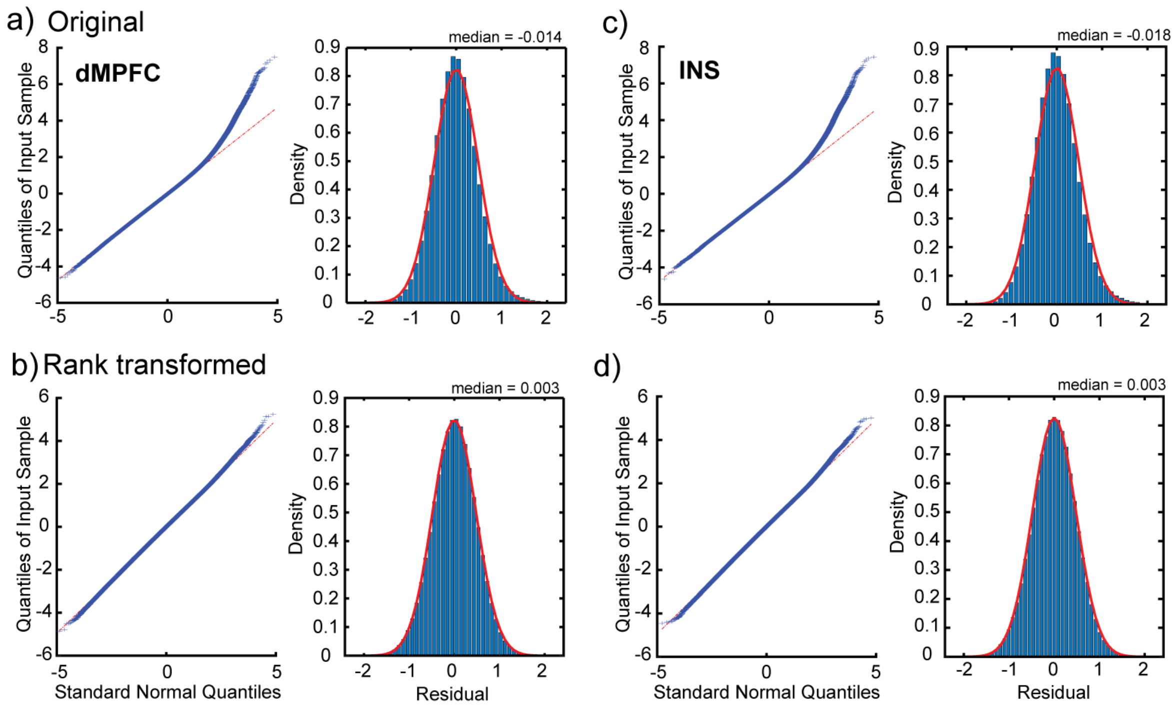

Supplementary Figure 4. ***Check of model assumptions for HFA residuals.*** *Quantile-quantile plots (left panels) and histograms (right panels) are used to inspect the distribution of residuals for dorsolateral prefrontal cortex (dMPFC; a,b) and insula (INS; c,d). Residuals are presented for both the original HFA data (top) and their rankit transformation (bottom). Red contours depict a reference normal distribution centered at 0. Residuals were scaled to their standard deviation. Histogram counts were transformed into probability densities. Source data can be reproduced using the open access data and code repositories.*

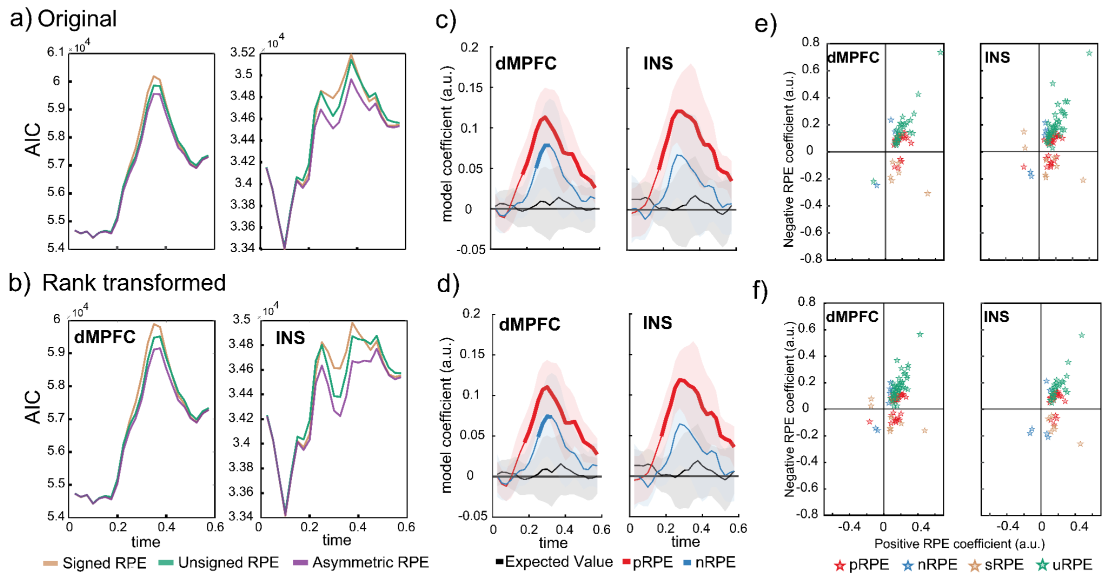

Supplementary Figure 5. ***Comparison between original (top) and sensitivity (bottom) analyses of HFA predicted by RPE features.*** *Convergent results are shown for model comparisons (**a, b**), asymmetric model coefficients (**c,d**) and peak coefficients for each channel colored per category (**e,f**). Statistics computed as describe in Fig. 2 and 3. Source data can be reproduced using the open access data and code repositories.*

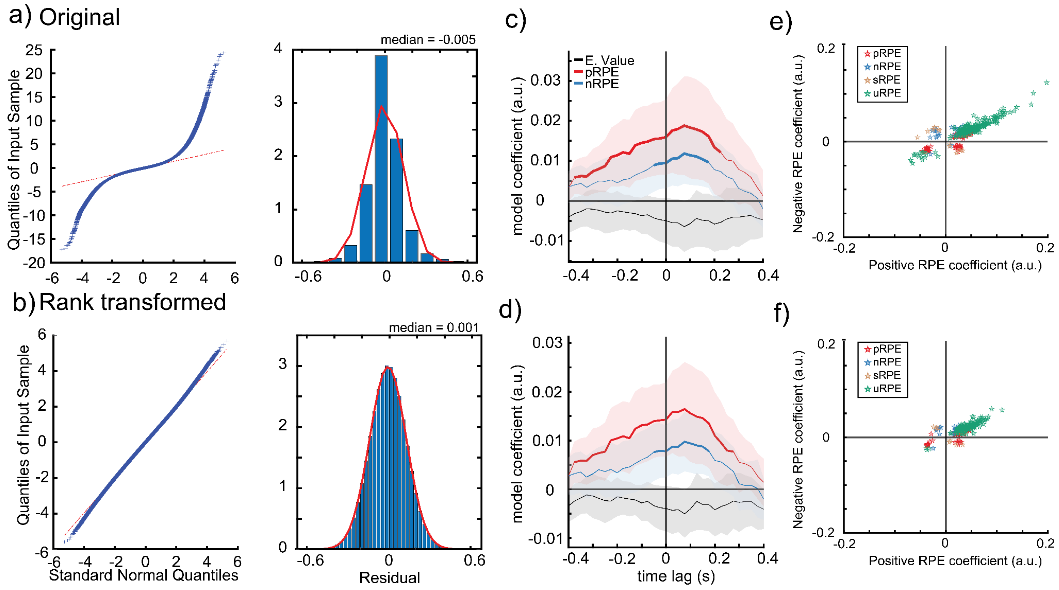

Supplementary Figure 6. ***Check of model assumptions and sensitivity analysis for HFA connectivity models.*** *(**a,b**) Quantile-quantile plots (left panels) and histograms (right panels) are used to inspect the distribution of residuals. Asymmetric model coefficients (**c,d**) and peak coefficients for each channel colored per category (**e,f**) are also shown. Results are presented for both the original HFA data (top) and their rankit transformation (bottom). Red contours depict a reference normal distribution centered at 0. Histogram counts were transformed into probability densities. Statistics as described in Fig. 4. Source data can be reproduced using the open access data and code repositories.*
