# Supplementary material for: Asymmetric coding of reward prediction errors in human insula and dorsomedial prefrontal cortex
Source: Nat Commun. 2023 Dec 21;14:8520. doi: 10.1038/s41467-023-44248-1 (PMC10739882; doi:10.1038/s41467-023-44248-1)
Supplement: Supplementary file 3 — Reporting Summary [file 41467_2023_44248_MOESM3_ESM.pdf]

nature portfolio

Corresponding author(s): Colin W. Hoy

Last updated by author(s): Oct 23, 2023

Reporting Summary

Nature Portfolio wishes to improve the reproducibility of the work that we publish. This form provides structure for consistency and transparency in reporting. For further information on Nature Portfolio policies, see our Editorial Policies and the Editorial Policy Checklist.

Statistics

For all statistical analyses, confirm that the following items are present in the figure legend, table legend, main text, or Methods section.

n/a	Confirmed	
☐	☒	The exact sample size (n) for each experimental group/condition, given as a discrete number and unit of measurement
☐	☒	A statement on whether measurements were taken from distinct samples or whether the same sample was measured repeatedly
☐	☒	The statistical test(s) used AND whether they are one- or two-sided<br>*Only common tests should be described solely by name; describe more complex techniques in the Methods section.*
☐	☒	A description of all covariates tested
☐	☒	A description of any assumptions or corrections, such as tests of normality and adjustment for multiple comparisons
☐	☒	A full description of the statistical parameters including central tendency (e.g. means) or other basic estimates (e.g. regression coefficient) AND variation (e.g. standard deviation) or associated estimates of uncertainty (e.g. confidence intervals)
☐	☒	For null hypothesis testing, the test statistic (e.g. F, t, r) with confidence intervals, effect sizes, degrees of freedom and P value noted<br>*Give P values as exact values whenever suitable.*
☒	☐	For Bayesian analysis, information on the choice of priors and Markov chain Monte Carlo settings
☐	☒	For hierarchical and complex designs, identification of the appropriate level for tests and full reporting of outcomes
☒	☐	Estimates of effect sizes (e.g. Cohen's d, Pearson's r), indicating how they were calculated

Our web collection on statistics for biologists contains articles on many of the points above.

Software and code

Policy information about availability of computer code

Data collection	Experimental task paradigm coded in PsychoPy v1.85.3 and run on Windows 10; Intracranial EEG data collected on Windows 10 computers using Nihon Kohden and Neuralynx recording software. For behavioral datasets from prior EEG study (Hoy et al., 2021), experimental paradigm was run and data was collected on Windows XP computer.
Data analysis	The Fieldtrip toolbox (version d073bb2de) and custom Python (2.7), R (2022.12.0+353, lme4 package 1.1.31), and MATLAB (2017b) code was used for preprocessing and analysis on a Macbook Pro (OS 10.13.6). All preprocessing, analysis, and plotting code is available as a GitHub repository (https://github.com/hoycw/asymmetric_RPE_paper) including system requirements and dependencies.

For manuscripts utilizing custom algorithms or software that are central to the research but not yet described in published literature, software must be made available to editors and reviewers. We strongly encourage code deposition in a community repository (e.g. GitHub). See the Nature Portfolio guidelines for submitting code & software for further information.

Data

Policy information about availability of data

All manuscripts must include a data availability statement. This statement should provide the following information, where applicable:

- Accession codes, unique identifiers, or web links for publicly available datasets
- A description of any restrictions on data availability
- For clinical datasets or third party data, please ensure that the statement adheres to our policy

The raw intracranial and anatomical datasets generated during the current study are not publicly available to preserve patient anonymity. The preprocessed behavioral and intracranial datasets generated and/or analyzed during the current study are available as a publicly repository in the Zenodo database (https://doi.org/10.5281/zenodo.10023443). The EEG datasets from healthy participants used for behavioral modeling are available in the Open Science Foundation repository and can be found at https://doi.org/10.17605/OSF. IO/JGXFR.

Human research participants

Policy information about studies involving human research participants and Sex and Gender in Research.

Reporting on sex and gender	Sex (n = 10, 1 female for intracranial patients; n = 32, 22 female for healthy participants from prior EEG study) was self-reported and not considered in the study design due to opportunistic sampling strategy. Disaggregated data is available in Table 1 for the intracranial patients and is described in Hoy et al. (2021) for the healthy participants. Sex- and gender-based analyses were not performed due to the lack of statistical power and imbalanced groups.
Population characteristics	Ten intracranial participants had a mean age of 32.5 (SD = 13.1 years), had normal IQ (>85), spoke fluent English, and were all undergoing neurosurgical treatment for epilepsy. The 32 healthy participants from the prior EEG study had a mean age of 20.7 (SD = 1.4 years), were recruited at the University of California, Berkeley, reported no history of psychiatric or neurological disorders, and had normal, or corrected-to-normal, vision.
Recruitment	Participants in the iEEG study were recruited from all patients undergoing intracranial monitoring for clinical treatment of epilepsy during the duration of the study that met the following inclusion criteria of being over 18 years old and English as a first language, meaning these results may not generalize beyond these populations. Only participants with electrode coverage in both dorsomedial prefrontal cortex and insula were included in this study due to a prior anatomical hypotheses. Due to these constraints, only one iEEG participant was female, meaning future studies should examine potential sex differences. Participants were informed about the possibility to participate in basic research before undergoing implantation of stereotactic EEG and electrocorticography electrodes for the localization of seizure foci. Informed consent was obtained from those still interested in participating after implantation of the electrodes. Details available for the healthy participants (undergraduate students with no reported history of psychiatric or neurological conditions) in the Reporting Summary for Hoy et al. (2021, Communications Biology).
Ethics oversight	University of California, Berkeley, University of California, Irvine, and California Pacific Medical Center Committees on Human Research

Note that full information on the approval of the study protocol must also be provided in the manuscript.

Field-specific reporting

Please select the one below that is the best fit for your research. If you are not sure, read the appropriate sections before making your selection.

☐ Life sciences ☒ Behavioural & social sciences ☐ Ecological, evolutionary & environmental sciences

For a reference copy of the document with all sections, see nature.com/documents/nr-reporting-summary-flat.pdf

Behavioural & social sciences study design

All studies must disclose on these points even when the disclosure is negative.

Study description	This study collected quantitative behavioral and intracranial electroencephalography (iEEG) data from 10 participants. Additional behavioral data collected from 32 healthy adults that performed the same task as part of a previous EEG publication (Hoy et al., 2021) are also analyzed.
Research sample	iEEG and behavioral data was collected from 10 adult participants with epilepsy (mean ± SD [range]: 35.2 ± 13.4 [21-57] years old; 1 woman) at the University of California, Irvine and California Pacific Medical Center hospitals. All participants reported no history of psychiatric disorders and had normal, or corrected-to-normal, vision. This sample is representative of normal cognitive function, as data with any epileptic activity was excluded from all analyses. Only participants with electrode coverage in both dorsomedial prefrontal cortex and insula were included in this study due to a prior anatomical hypotheses. All possible participants the fit the inclusion criteria for the study were recruited to maximize the sample size of these rare data. The experiment was designed to

compare conditions within each subject to allow inference with a relatively small sample.

For the healthy participants in the prior EEG study (Hoy et al. 2021, Communications Biology), data was sought from ~30 participants to exceed the standard sample size in EEG research experiments. Furthermore, the experiment was designed to collect large numbers of trials in each participant for rare conditions (e.g., 600 trials per participant yields 72 trials for rare neutral outcomes on 12% of trials), thereby providing unusually large statistical power for an EEG study even after allowing for artifact rejection.

Sampling strategy	Due to the rare nature of these data, a convenience sample was recruited from all potentially eligible patients being treated for epilepsy at the two hospitals. Participants in the iEEG study were required to meet the inclusion criteria of being over 18 years old and English as a first language. Sample size is adequate due to the high signal-to-noise characteristics of iEEG data and the strong statistical power provided by single-trial computational modeling using hierarchical linear mixed effects models. See Reporting Summary for Hoy et al. (2021, Communications Biology) for details on recruitment of healthy participants from prior EEG study.
Data collection	The data were recorded at either the University of California Irvine Medical Center (n = 9), USA or CalFifornia Pacific Medical Center (n = 1), USA. Patients at Irvine were implanted with stereo-EEG (SEEG) electrodes with 5 mm spacing, and the patient at CPMC was implanted with strips of electrocorticography (ECoG) electrodes with 1 cm spacing. At both sites, electrophysiology and analog photodiode event channels were recorded using a 256-channel Nihon Kohden Neurofax EEG-1200 recording system and sampled at 500 (n = 3), 1000 (n = 3), or 5000 Hz (n = 4). For five patients, analog photodiode channels and a subset of iEEG channels were recorded in a separate Neuralynx ATLAS recording system at Irvine at 4000 (n = 1) or 8000 Hz (n = 4). For these cases, photodiode events were then aligned to the iEEG data acquired in parallel via the Nihon Kohden clinical amplifier via cross-correlation of shared iEEG channels. Pre-operative T1 MRI and post-implantation CT scans were collected as part of standard clinical care and used to determine recording locations. All participants but not experimenters were blinded to the study hypothesis. Participants were not blinded to the difficulty of the blocks. During some data recording sessions, family members of the patients were in the room during iEEG data collection, in which case those family members were instructed to remain silent and not distract the patient. Trials with distractions and/or interruptions were removed during preprocessing. See Reporting Summary for Hoy et al. (2021, Communications Biology) for details on data collection from healthy participants from prior EEG study.
Timing	Intracranial data were collected continuously from March 2017 to February 2020. Healthy participant data were collected for the prior EEG study from October 2018 to February 2020.
Data exclusions	Raw iEEG traces were manually inspected by a neurologist for epileptic spiking and spread, as well as artifacts (e.g., machine noise, signal drift, amplifier saturation, etc.). Data in regions or epochs with epileptiform or artifactual activity were excluded from further analyses. After preprocessing (see Methods), continuous data were then visually inspected to ensure all epochs with artifacts or spread from epileptic activity were removed. Finally, trials were rejected for task interruptions and behavioral outliers (RTs missing, < 0.5 s, > 1.5 s, or > 3 standard deviations from that patient's mean), resulting in 274-890 trials per patient (mean ± S.D.: 405.0 ± 210.6). Behavioral exclusion criteria were pre-established based on prior studies (Hoy et al., 2021 Communications Biology), and neural exclusions were not pre-determined due to the idiosyncratic nature of epilpetic activity and noise profiles across participants and recordings. See Reporting Summary for Hoy et al. (2021, Communications Biology) for details on data exclusions for healthy participants from prior EEG study.
Non-participation	No participants dropped out or declined participation.
Randomization	Participants were not allocated into groups due to the within-subjects experimental design in which all participants performed all conditions. Note that the random neutral feedback condition was introduced in a later experimental design, which was used for the last four participants recruited.

Reporting for specific materials, systems and methods

We require information from authors about some types of materials, experimental systems and methods used in many studies. Here, indicate whether each material, system or method listed is relevant to your study. If you are not sure if a list item applies to your research, read the appropriate section before selecting a response.

Materials & experimental systems

n/a	Involved in the study	
☒	☐	Antibodies
☒	☐	Eukaryotic cell lines
☒	☐	Palaeontology and archaeology
☒	☐	Animals and other organisms
☒	☐	Clinical data
☒	☐	Dual use research of concern

Methods

n/a	Involved in the study	
☒	☐	ChIP-seq
☒	☐	Flow cytometry
☒	☐	MRI-based neuroimaging
